# 4Ms-Behavioral Health in Australia: An International Comparison

**DOI:** 10.1093/geroni/igaf122.652

**Published:** 2025-12-31

**Authors:** Erin Emery-Tiburcio, Laura Porter

**Affiliations:** Rush University Medical Center, Chicago, Illinois, United States; Cherokee Health Systems, Knoxville, Tennessee, United States

## Abstract

The 4Ms framework of an Age-Friendly Health System (What Matters, Medication, Mentation, Mobility) was originally developed by the Institute for Healthcare Improvement with support from The John A. Hartford Foundation for hospital and ambulatory care settings. As many behavioral health providers did not clearly see a role for themselves in all 4Ms, the 4Ms-Behavioral Health framework (4Ms-BH) was adapted to clarify roles and include additional critical elements for older adult mental health and substance use. Following piloting in the US, the 4Ms-BH training was tailored for Australian audiences with language, cultural, and health system references. Individual interviews with Australian health providers indicated that the training was most needed in inpatient health settings, thus the training was adapted further to meet these needs. The training has been provided to interprofessional teams at a rural hospital in Cairns, Queensland (n = 49), and an urban hospital in Gold Coast, Queensland (n = 25). Initial qualitative data analysis indicates that these Queensland health systems are far ahead of most US health systems in their screening for and addressing delirium, obtaining basic advance directives, and incorporating pharmacy in attending to polypharmacy and high risk medications. Critical elements internationally are widespread ageism and connecting the 4Ms as a set. Discussion will focus on unique cultural elements and international similarities in 4Ms-Behavioral Health implementation.

